# Can a tablet-based cancellation test identify cognitive impairment in older adults?

**DOI:** 10.1371/journal.pone.0181809

**Published:** 2017-07-24

**Authors:** Ya-Huei Wu, Jean-Sébastien Vidal, Jocelyne de Rotrou, Sietske A. M. Sikkes, Anne-Sophie Rigaud, Matthieu Plichart

**Affiliations:** 1 Assistance Publique – Hôpitaux de Paris, Hôpital Broca, Paris, France; 2 Paris Descartes University, Sorbonne Paris Cité, Paris, France; 3 Alzheimer Center, VU University Medical Center, Amsterdam, the Netherlands; 4 Department of Epidemiology and Biostatistics, VU University Medical Center, Amsterdam, the Netherlands; University of California, San Francisco, UNITED STATES

## Abstract

**Background and objective:**

There has been a growing interest in using computerized cognitive assessment to detect age-related cognitive disorders. We have developed a tablet-based cancellation test (e-CT), previously shown as a reliable measure of executive functions and free of effect of familiarity with computer-based devices in healthy older adults. This study aimed to investigate the influence of demographics and current daily use of computer-based devices in older adults with Mild Cognitive Impairment (MCI) and Alzheimer’s disease (AD). We further studied the ability of the e-CT to discriminate MCI and AD patients from older adults with normal cognition (NC).

**Methods:**

The e-CT was administered to 325 older adults (NC = 112, MCI = 129, AD = 84). Subjects also performed the K-T test, a paper-and-pencil cancellation test from which the e-CT was developed. Multiple linear regression analyses were conducted to assess the contribution of demographics and current daily use of computer-based devices on the e-CT in patient groups. The Receiver Operating Characteristic (ROC) curves and the Area Under the Curve (AUC) were established to compare the efficacy of the e-CT and the K-T test to classify subjects into diagnostic groups.

**Results:**

In the MCI group, age (*B* = -0.37, p<0.001) and current daily use of computer-based devices (*B* = 5.85, p<0.001) were associated with the number of correct cancellations of the e-CT. In the AD group, only current daily use of a computer-based device was a significant contributor (*B* = 6.28, p<0.001). The e-CT (AUC = 0.811; 95% confidence interval [CI]: 0.756–0.867) and the K-T (AUC = 0.837; CI: 0.787–0.887) showed good and comparable diagnostic accuracy to discriminate between MCI and NC subjects. To discriminate between NC and AD, both tests showed high diagnostic accuracy, with the AUC values of 0.923 (CI: 0.876–0.971) and 0.929 (95%CI: 0.886–0.972) for the e-CT and the K-T, respectively.

**Conclusion:**

The e-CT presents satisfying discriminative validity and is a promising tool for detection of early cognitive impairment in older adults.

## Introduction

In spite of the evidenced potential of biomarkers to identify the Alzheimer’s disease (AD) pathophysiological process, cognitive tests remain essential for the diagnosis of AD, even in its preclinical stages (i.e., Mild Cognitive Impairment; MCI) [1, 2]). Measuring executive functions (EF) in early stage of AD is relevant since it was shown that AD patients present various degrees of impairment of EF [3]. Furthermore, the latest published criteria for “MCI due to AD” emphasized that along with episodic memory impairment, other cognitive domains, including EF, are also impaired in MCI patients who progress to AD [4]. EF are multifaceted and encompass a set of abilities central to human adaptive behaviors and allow one to engage in goal-directed behaviors [5]. These abilities include planning, problem solving, abstracting, strategy development and implementation, working memory, inhibitory control and mental flexibility. It is believed that these abilities are essential to function successfully in everyday living [6, 7]. Increasing evidence shows that MCI patients have impaired performances on several EF measures, such as Trail-Making Test, verbal fluency, Backward Digit Span, Stroop, etc. and suggests that measures of EF predicted the progression from MCI to AD [8–10].

Recently, there has been a growing interest in using computerized cognitive assessment to detect age-related cognitive disorders [11, 12]. The computerized cognitive assessment tools present several core features, such as the standardization of test administration, automatic recording of several parameters of the responses (time, latency and variability) and automatic scoring [13, 14], which reduce or even eliminate the examiner effects and human errors during the administration, data entry and scoring [2, 15]. These features are especially interesting for the measure of EF, during which a test taker’s performance is reflected by reaction time and/or responses (correct hits and errors). Almost all computerized cognitive batteries include EF measures. Most of them are adapted from traditional paper-and-pencil tests. For instance, the Stroop test, the Finger Tapping test, the Spatial Span test and the Go-No Go test were adapted to various computerized versions in batteries, such as CAMCI, CANS-MCI, CANTAB, CNS-Vital Signs and Mindstreams. However, individual tests in these batteries were not validated.

We have developed a tablet-based cancellation test (e-CT), adapted from a paper-and-pencil cancellation test (K-T cancellation test) [16]. Cancellation tests are commonly used to assess a person’s ability to simultaneously target stimuli while ignoring distractors. During the tasks, subjects are asked to cross out target stimuli as quickly as possible. The subject’s performance is assessed by different criteria, e.g., the number of correctly and incorrectly identified target stimuli, as well as the time spent to complete the task. Successful performances on CTs require sustained and selective attention, visuospatial search, psychomotor speed, and fine motor coordination [17–19]. One of the most characteristic features of CTs is that they are administered in a self-paced manner. This implies that individuals have to internally monitor their own speed-accuracy balance, which is considered as a resource-demanding process requiring the implementation of supervisory executive functions [20, 21]. Therefore, fundamental executive mechanisms, such as planning, organizing, selecting relevant pieces of information, and inhibiting irrelevant ones are needed when performing these tests. Various versions of cancellation tests have been used to discriminate healthy older adults from those who suffer from early dementia [22–24].

Previously, in a sample of healthy older adults, we demonstrated that the e-CT shows good test-retest reliability and satisfying convergent validity with traditional measures of EF. Furthermore, the e-CT was sensitive to aging effects, but that performance was not influenced by sex, education and familiarity with computer-based devices [25].

As a part of psychometric validation of the e-CT, this study aimed to investigate its clinical utility. Potential influencing factors, especially current daily use of computer-based devices were investigated in MCI and AD patients. We compared the performance on the e-CT in older adults with normal cognition (NC), patients with MCI and patients with AD. We further compared the efficacy of the e-CT and K-T cancellation test to discriminate NC, MCI and AD subjects.

## Materials and methods

### Study participants

The participants were recruited from a Memory Clinic between July 2013 and November 2015 and received a comprehensive geriatric assessment including a complete physical examination, biological analyses (measurement of thyroid function, vitamin B12 and folate levels, natremia, calcemia, etc.) and an assessment of cognitive function and of functional status in daily life. For most of them, MRI measures of hippocampal atrophy were obtained.

AD patients were diagnosed according to the criteria of National Institute of Neurological and Communicative Disorders and Stroke and Alzheimer Disease and Related Disorders Association [26]. These subjects showed impairment in memory plus other cognitive domains and in activities of daily living.

The subjects were diagnosed with MCI according to Petersen criteria [27]. They were non-demented, had preserved functional abilities and performed at or below 1.5 standard deviations below the mean for age and education, according to published norms, on more than one of the neuropsychological tests.

For this study, we used a convenience sample. The participants were considered to be cognitively normal if they 1) did not present any cognitive impairment as measured by the neuropsychological test battery from the diagnostic unit; 2) did not present a history of neurological and psychiatric disorders and 3) did not present any functional impairment.

The exclusion criteria for all the participants were as follows: 1) the presence of motor or visual abnormalities affecting the performance on neuropsychological tests; 2) the presence of psychiatric or neurological disorders which could cause cognitive impairment; 3) a history of alcohol or other substance abuse.

The diagnoses were made by geriatricians, blinded to the results of the K-T cancellation test and the e-CT test.

The study was approved by the Paris Descartes University Institutional Review Board (CERES). Informed consents were obtained from all the participants.

### Measures and procedure

#### Neuropsychological assessment

Global cognitive function was evaluated by the Mini Mental State Examination (MMSE) [28] as part of the neuropsychological assessment. Executive abilities were assessed using a variety of measures including: the Digit Span [29], Trail Making Test part A and B [30], letter word list generation (P, 2 minutes) and semantic category fluency (animals, 2 minutes). Episodic memory was assessed by the French version of Free and Cued Selective Reminding Test (FCSRT): Rappel Libre/Rappel Indicé à 16 items (RL/RI-16) [31, 32], and 3 subtests of Cognitive Efficiency Profile [33]: logical memory with two free recalls of a narrative story and the visuo-spatial memory test with the reproduction of a complex figure.

#### K-T cancellation test [34]

**Fig 1:**
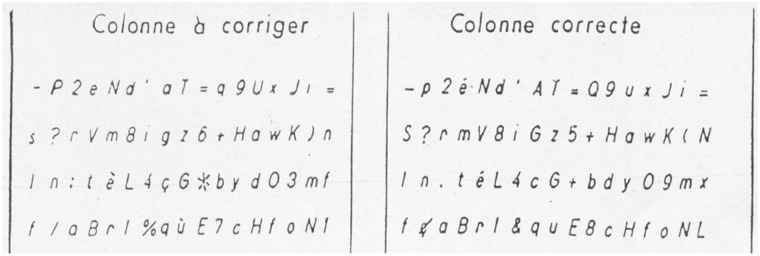
K-T cancellation test.

Two blocks (one on the right hand side and the other one on the left hand side) of stimuli composed of letters, numbers and symbols are printed on an A3-size sheet of paper. Each block consists of 340 stimuli (20 lines of 17 stimuli) (Fig 1). Participants were asked to cross out with a pencil on the left-side block the stimuli that were not identical with those on the right-side block. The participants were asked to complete the test from left to right and from top to bottom as rapidly and accurately as possible. In total, there are 117 stimuli to be crossed out. The time allocated to complete the test was 3 minutes. The participants were asked to draw a bracket on the last stimulus processed when the administrator told them to stop. Scoring consists of the number of correct cancellations, omission errors and commission errors [34,35].

#### e-CT

**Fig 2:**
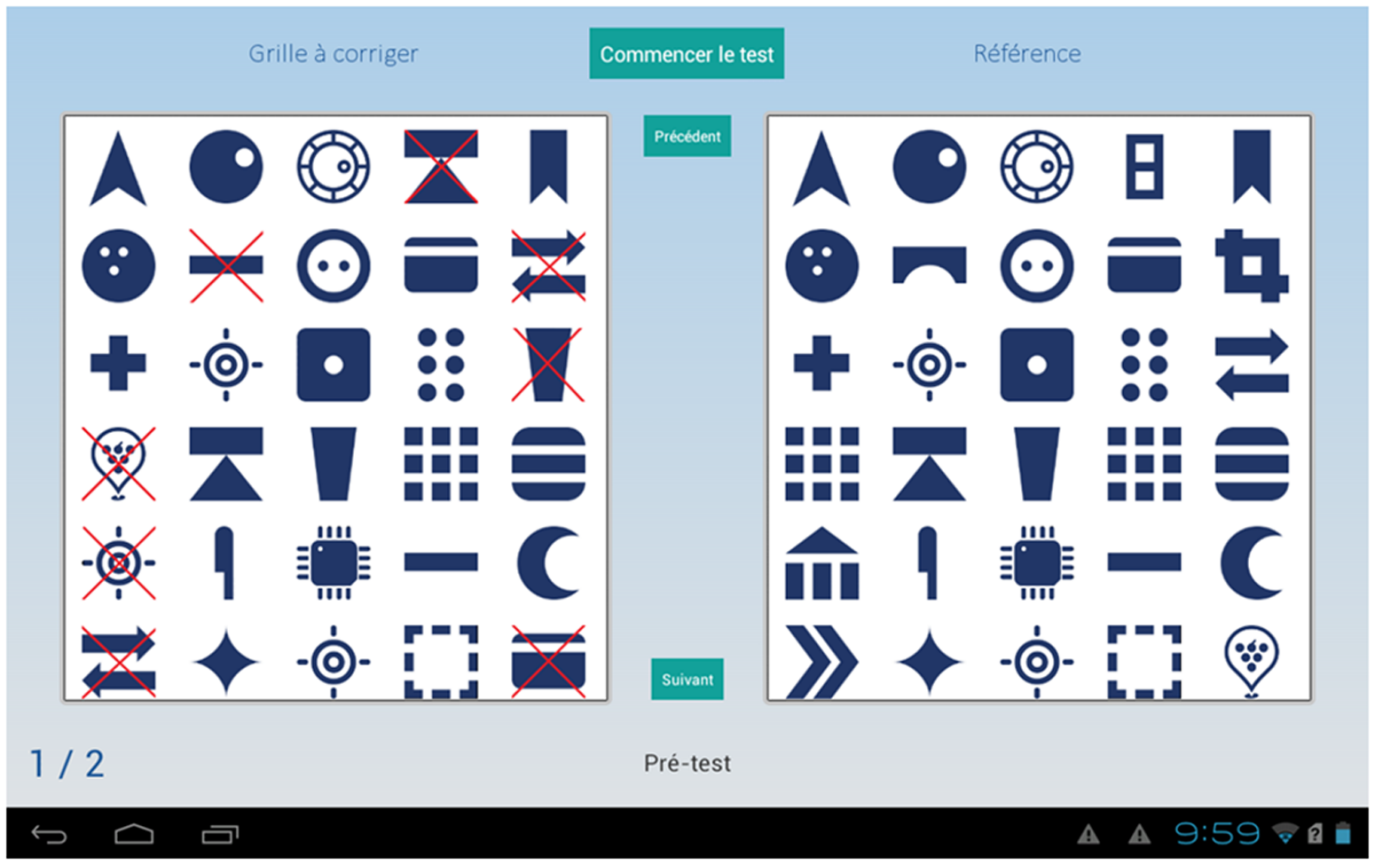
Table-based cancellation test (e-CT).

The e-CT (Fig 2) was developed and adapted from the K-T cancellation test. The e-CT application was developed for Android tablets using Android Software Development Kit in Java language and was optimized for compatibility and performance of different devices.

Some changes from the K-T cancellation test were implemented when designing the computerized version, mainly due to the fact that a tablet screen cannot display as many stimuli as an A3-size paper sheet. We also used different stimuli in the e-CT; only pictograms were implemented.

Two blocks of stimuli composed of 30 symbols (6 lines of 5 stimuli) are displayed on the right and on the left of a touch screen (10.1 inches) of a tablet PC. The participants were asked to touch the stimuli on the left-side block that are not identical with those on the right-side block. They were also asked to complete the test from left to right and from top to bottom as rapidly and accurately as possible. Once participants finished processing all stimuli on a page screen, they must touch the “next” button to continue the test. In total, there are 7 pages of stimuli and 62 stimuli are to be crossed out. The time allocated to complete the test was 2 minutes.

To start the test, the participants must touch a “Start” button and after 2 minutes, the system stops the test automatically by displaying on a new screen page the test results: the number of correct cancellations, omission errors and commission errors.

To get the participants familiarized with the tablet PC, a practice session with 2 pages of stimuli was proposed. During the practice session, the psychologists gave explanations when necessary in order to ensure that all the participants understood the test instructions and how the tablet PC works. During the test session, they also recorded how many times the participants needed to be reminded to touch the “next” button to process a new page of stimuli (instruction reminder).

Subjects performed the e-CT and the K-T cancellation test (K-T), in a different session (but on the same day) than that of the neuropsychological assessment. The order of administration of the two versions of CT was randomized.

#### Measurement of current daily use of computer-based devices

The participants reported how often they currently used a computer and a tablet PC/Smartphone/PC. They were classified as either daily users (use any aforementioned computer-based devices at least once a day) or non-daily users.

### Statistical analysis

Chi-square (*X*^2^) tests were used to compare sex, current daily use of computer-based devices (daily users vs. non-daily users) and educational level (>12 years vs. ≤12 years) across the groups (NC vs. MCI vs. AD). One-way between-subjects analysis of variance (ANOVA) was performed to determine whether groups differed significantly with regards to age and MMSE score. Post-hoc analyses (Bonferroni correction) were performed in cases of a significant group effect to determine the differences between the groups.

Multiple linear regression analyses were conducted separately for MCI and AD groups, in order to assess the relative contribution of age, education, sex, and current daily use of computer-based devices on the number of correct cancellations of the e-CT.

The correct cancellations of e-CT were compared among diagnostic groups using one-way analysis of covariance (ANCOVA) in which demographics (age, sex, education) and current daily use of computer-based devices were treated as factors and covariates. Bonferroni post-hoc tests were conducted for pairwise multiple comparisons. Omission errors, commission errors and the number of instruction reminders of the e-CT were recoded as binary variables (0 vs. ≥1 errors/time). Chi-square tests were used to identify error rate differences across the diagnostic groups.

We developed demographically corrected T scores on the basis of the regression method of Heaton et al. [36]. Influences of age, education, sex, current use of computer-based devices on correct cancellations of the e-CT and the K-T test were assessed by performing multiple linear regression analyses for the group of healthy older adults. From the multiple linear regression equations, only variables significant at p<0.05 were retained. The intercept and unstandardized regression coefficients and square root of mean square residual estimated from the healthy older adults were used to calculate predict scores for the e-CT and the K-T test in MCI and AD patients. Predicted scores were subtracted from obtained scores to calculate residual scores, which were converted to T scores according to the following formula:

T score=[(residual score/square root of mean square residual)×10]+50

The Receiver Operating Characteristic (ROC) curves and the Area Under the Curve (AUC) were established to compare the diagnostic performance of T scores of correct cancellations of e-CT and of K-T to discriminate between the NC subjects and the cognitively impaired patients. An AUC value greater than 0.9 indicates high accuracy, > 0.8 indicates good accuracy, > 0.7 indicates fair accuracy and > 0.6 indicates poor accuracy[37]. Optimal cut-off scores and associated values of sensitivity (Se) and specificity (Sp) were also calculated.

All statistical analyses were performed using IBM SPSS Statistics 17 for Windows.

## Results

### Sample characteristics

Results were obtained from 325 participants, aged between 50 and 92 years, with a mean of 75.9 (SD = 6.96). There were 112 men (34.5%) and 213 women (65.5%). One hundred and eighty- eight (57.8%) had a higher level of education (>12 years). One hundred and ninety-five (60%) used a computer-based device on a daily basis. The sample was composed of 112 NC subjects, 129 MCI patients and 84 AD patients. Table 1 shows the characteristic of the three groups. There was no significant group difference on age. Compared to patient groups, there was significantly higher proportion of women and subjects with higher education level in the NC group. MMSE score and the percentage of subjects using a computer-based device daily decreased with increasing cognitive impairment.

**Table 1:** Characteristics of NC, MCI and AD groups and their performance on the e-CT.

	NC (N = 112)	MCI (N = 129)	AD (N = 84)	p value
**Demographics**				
Age, years, mean (SD)	74.7 (6.94)	76.5 (7.49)	76.5 (5.94)	0.081*
Male, n (%)	28 (25.0)	48 (37.2)	36 (42.9)	0.024†^1^^,^^2^
Education>12 years: n (%)	79 (70.5)	64 (49.6)	45 (53.6)	0.003†^1^^,^^2^
Daily computer-based technology use: n (%)	88 (78.6)	75 (58.6)	32 (38.1)	<0.001†^1^^,^^2^^,^^3^
MMSE	28.9 (1.01)	27.1 (2.30)	23.5 (3.10)	<0.001*^1^^,^^2^^,^^3^
**e-CT**				
Correct cancellations, mean (SD)	40.2 (5.43)	31.1 (9.00)	24.3 (8.72)	<0.001#^1^^,^^2^^,^^3^
Omission errors (≥ 1): n (%)	72 (64.9)	74 (57.8)	47 (56.0)	0.385†
Commission errors (≥ 1): n (%)	20 (17.9)	25 (19.5)	21 (25.0)	0.449†
Instruction reminder (≥ 1): n (%)	6 (5.4)	20 (15.6)	36 (42.9)	<0.001†^1^^,^^2^^,^^3^

*ANOVA test

^†^Chi-square test

^#^ANCOVA test

NC, normal cognition; MCI, mild cognitive impairment; AD, Alzheimer’s disease

^1:^ NC ≠ MCI;

^2:^ NC ≠ AD;

^3:^ MCI ≠ AD

### Effect of demographics and current daily use of computer-based devices on correct cancellations of e-CT in MCI and AD groups

In the MCI group, age (*B* = -0.37, p<0.001) and current daily use of a computer-based device (*B* = 5.85, p<0.001) were associated with the number of correct cancellations. In the AD group, only current daily use of a computer-based device was significantly associated with the number of correct cancellations (*B* = 6.28, p<0.001). In both MCI and AD groups, daily users of a computer-based device outperformed non-daily users on the e-CT.

An additional analysis showed that non-daily users of computer-based technology also performed worse on K-T than daily users among MCI (F_1, 119_ = 9.64, p = 0.002, η^2^_p_ = 0.075) and AD subjects (F_1, 74_ = 12.7, p = 0.001, η^2^_p_ = 0.146), after controlling for education, age and sex.

### Comparison among diagnostic groups on e-CT

NC subjects had more correct cancellations on the e-CT (M = 40.2, SD = 5.43; range = 18–56) than MCI patients (M = 31.1, SD = 9.00; range = 11–50) who outperformed the AD patients (M = 24.3, SD = 8.72; range = 6–51). After adjusting for demographics and current daily use of a computer-based device, the differences among the groups remained significant, F (2, 317) = 81.2, *p* < 0.001, η^2^_p_ = 0.339.

In comparison with the NC group, a higher proportion of patients in the MCI group (*X*^2^(1) = 6.52, p = 0.011) needed to be reminded of the instructions and this variable also differentiated MCI and AD groups (*X*^2^(1) = 19.4, p < 0.001).

There were no significant differences between the groups regarding the proportion of subjects making ≥ 1 omission errors (*X*^2^(2) = 1.91, p = 0.385) and the proportion of subjects making ≥ 1 commission errors (*X*^2^(2) = 1.60, p = 0.449) (Table 1).

### Comparison of the efficacy between e-CT and K-T to classify subjects into diagnostic groups

In the NC group, it was found that only age was related to performance on the e-CT and the K-T test. Therefore, regression equations were built that used age as predictor. Table 2 presents the conversion of obtained scores of correct cancellations to age corrected T scores for e-CT and the K-T.

**Table 2:** Conversion of obtained scores to age-corrected T scores for e-CT and K-T.

	values obtained from regression analyses		predict score	Z score	T score
e-CT	*B* for intercept	75.220	75.220-(0.468 × age)	(raw score-predicted score)/4.366	50+ (10×Z)
	*B* for age	-0.468			
	Square root of mean square residual	4.366			
K-T	*B* for intercept	102.869	102.869-(0.721 × age)	(raw score-predicted score)/8.415	
	*B* for age	-0.721			
	Square root of mean square residual	8.415			

**Fig 3:**
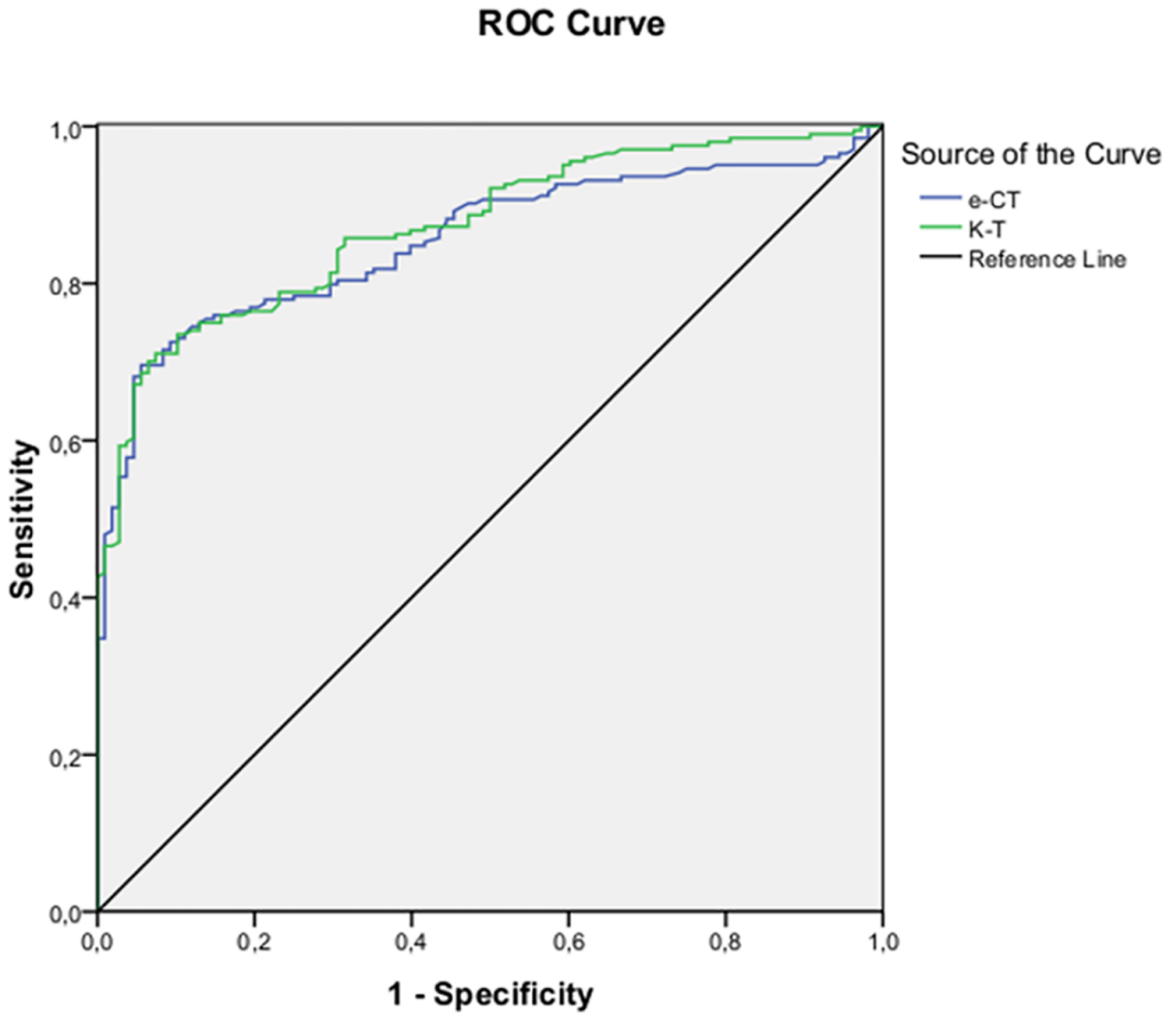
Receiver Operating Characteristic curves for the e-CT and the K-T discriminating subjects with cognitive impairment (MCI and AD) from cognitively normal older adults.

ROC curves were generated to compare the efficacy of e-CT and of K-T to discriminate between subjects with cognitive impairment (MCI + AD) and NC subjects (Fig 3). The value of AUC was 0.855 for e-CT and 0.873 for K-T.

**Fig 4:**
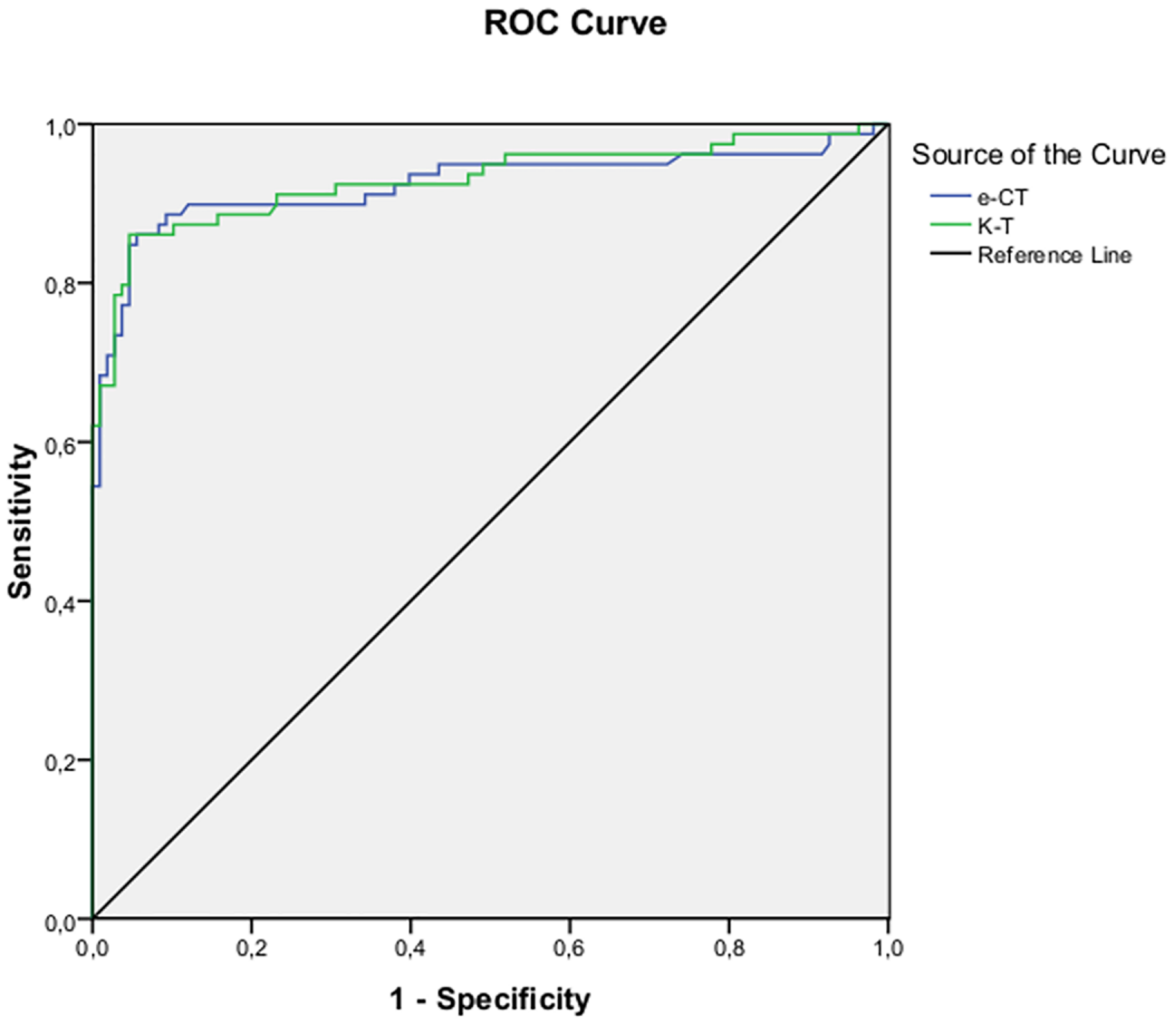
Receiver Operating Characteristic curves for the e-CT and K-T discriminating AD patients from cognitively normal older adults.

Both e-CT (AUC = 0.923) and K-T (AUC = 0.929) had a high discriminative ability to distinguish NC subjects from AD patients (Fig 4).

Table 3 shows values of AUC, Se, Sp and optimal cut-off scores of e-CT and K-T to discriminate between the diagnostic groups.

**Table 3:** Discrimination between diagnostic groups for the e-CT and the K-T.

Diagnostic group	Test	AUC	Sensitivity	Specificity	Optimal cutoff score
NC vs. MCI+AD	e-CT	0.855 (CI:0.813–0.896)	0.770	0.806	41.52
	K-T	0.873 (CI:0.835–0.910)	0.765	0.806	40.95
NC vs. AD	e-CT	0.923 (CI:0.876–0.971)	0.861	0.917	36.36
	K-T	0.929 (CI:0.886–0.972)	0.861	0.944	36.26
NC vs. MCI	e-CT	0.811 (CI:0.756–0.867)	0.704	0.787	41.91
	K-T	0.837 (CI:0.787–0.887)	0.712	0.769	41.93

NC, normal cognition; MCI, mild cognitive impairment; AD, Alzheimer’s diseases

## Discussion

This study addressed the clinical utility of the e-CT, a tablet-based cancellation test, previously validated as a measure of EF. Current daily use of a computer-based device was found to influence e-CT performance only in MCI and AD patient, but not in healthy older adults. Concerning its psychometric properties, e-CT presented good to high diagnostic accuracy for discriminating patients with MCI and AD from healthy older adults.

In our sample, daily computer-based technology use was different across the groups. The proportion of older adults using a computer-based device daily decreased with increasing cognitive impairment, suggesting a positive relationship between computer use as mentally stimulating activities and cognitive performance [38–41]. Lack of daily usage of computer-based devices in patient groups could be explained by cognitive impairment. Indeed, cognitive impairment can lead to difficulties using complex and changing interfaces [42].

In the NC group, the number of correct cancellations of the e-CT was influenced only by age, but not by current daily use of computer-based devices. This concurs with the study of Hansen et al. showing that there was not significant association between participants’ self-reported computer familiarity and performance on web-based tests, after adjusting for age [43]. However, in both patient groups (MCI and AD), daily use of a computer-based device was related to better performance on the e-CT. This implies that cognitively intact older adults, who did not use a computer-based device daily, could benefit from the practice session to acquire the skills necessary to perform a computer-based cognitive test. However, this was not the case for cognitively impaired patients, who did not use a computer-based device on a daily basis. These subjects needed more practice to get familiar with the tablet PC in order to perform e-CT. Another hypothesis is that in patient groups, those who did not use a computer-based device daily might be more impaired cognitively, compared to the daily users. This hypothesis is supported by a study showing that less computer activity is associated with poor executive control [38] and confirmed by an additional analysis of our data showing that in patient groups, non-daily users also performed worse on K-T than daily users.

The number of correct cancellations of the e-CT discriminated healthy elderly from MCI and AD patients, after adjusting for demographic variables and current daily use of a computer-based device, suggesting underlying deficits of alternation/flexibility and inhibition/interference control in patients groups. Furthermore, as cognitive impairment increased, a higher proportion of subjects needed to be reminded of the instructions to perform the e-CT, partly due to a failure of goal-maintenance and partly due to memory deficit. Finally, the errors of the e-CT did not differentiate the diagnostic groups. Our findings support the usefulness of various versions of cancellation tests, such as Zazzo’s cancellation test, to discriminate healthy older adults from those with dementia [22–24]. In MCI and AD patients, pathologic changes in the temporal lobes might impair the anatomic and functional connections between the prefrontal cortex (PFC) and temporal cortex, leading to the inefficacy of the PFC in EF tasks [44]. Alternatively, EF impairment could be explained by a disruption of frontal-subcortical pathways due to white matter lesions, a commonly observed brain pathology in the elderly with cognitive impairment [45, 46].

The diagnostic accuracy of correct cancellations of the e-CT was high to distinguish healthy elderly from AD patients and good to discriminate healthy elderly from MCI. Compared with other computer-based cognitive tests assessing EF, the values of diagnostic performance of the e-CT were greater than those of the attention/psychomotor composite of the CogState (AUC = 0.73 for discrimination between NC and AD; AUC = 0.67 for discrimination between NC and MCI) [47] and that of problem solving of the Mindstreams (AUC = 0.685 for discrimination between NC and MCI) [48]. The diagnostic values of the e-CT were comparable, though slightly lower than those of K-T, the paper-and-pencil cancellation test, from which e-CT was developed. These findings suggest that the e-CT is as discriminative as the K-T for detecting cognitive impairment. However, the e-CT has some advantages, such as automatic recording of time and scoring, which allows time-saving and error reduction during scoring.

There were some limitations in the study. Owing to the cross-sectional nature of the study, the etiologies of MCI patients without longitudinal follow-up are not clear. In addition, biomarker data were not obtained, thereby limiting the comparison between biomarkers and e-CT performance. Future studies could address these issues in order to understand the relationship between the e-CT performance and brain pathologies and to study the predictive value of e-CT in the conversion to dementia in MCI patients. Furthermore, as there were fewer men and subjects with lower educational level in the NC group, our findings might not be generalized. Future studies could compare clinical populations with more a representative sample of healthy older adults. Finally, computer-based device use was measured only by a self-report Likert scale questionnaire on frequency of use, which could not capture detailed information about the types of activities performed (communication, entertainment, banking, shopping, health information, etc.) on these devices. As a consequence, in the present work, the measure of current use of computer-based devices might not fully reflect one’s skills on these devices.

In conclusion, the present study demonstrated satisfying discriminative validity of the e-CT. Our previous study has presented other psychometric properties of the e-CT: convergent validity with EF measures and excellent test-retest fidelity. Altogether, these findings suggest the e-CT is a promising tool to detect early cognitive impairment in older adults and can be used to track EF changes over time in longitudinal studies or in clinical trials.
